# Personalized cancer vaccines: promise, challenges, and the path forward

**DOI:** 10.1097/MS9.0000000000003991

**Published:** 2025-09-30

**Authors:** Muskan Shafique, Karan Chaman Lal, MD Faisal Ahmed

**Affiliations:** a Department of Medicine, Sahiwal Medical College, Sahiwal, Pakistan; b Department of Medicine, Liaquat University of Medical & Health Sciences, Jamshoro, Sindh, Pakistan; c Department of Health Science and Informatics, Bangladesh Institute of Innovative Health Research, Dhaka, Bangladesh

*To the Editor,*

This study strictly adheres to the TITAN Guidelines and emphasizes that ‎other studies should follow this[1].

Personalized cancer vaccines are rapidly becoming one of modern oncology’s most hopeful developments. They mark a turn away from one-size-fits-all treatments and toward care designed for one person alone. Recent progress has been striking – for example, an mRNA-based personalized vaccine, given together with pembrolizumab in patients with resected melanoma, has significantly prolonged the time without recurrence[2]. Such results underscore the very real potential of this strategy.

Several vaccine types – including those built from peptides, mRNA, or patients’ dendritic cells – are now being studied. Early clinical trials in glioblastoma and pancreatic cancer have shown encouraging immune responses and reassuring safety profiles[3]. These personalized treatments are meticulously designed to target the unique protein fragments “neoantigen” arising from somatic cell mutations specific to an individual’s tumor.

One of the biggest advantages of such vaccines is their high specificity and work by eliciting an immune response directly against tumor cells, thereby minimizing the destruction of healthy tissue and reducing off-target effects. Furthermore, these vaccines hold significant promise for preventing disease recurrence and metastasis by generating long-term immunological memory. Moreover, they can reverse tumor-induced immunosuppression and enhance T-cell activation due to their synergy with immune checkpoint inhibitors[4].

Despite such notable potential, considerable challenges persist. Producing each vaccine is a complex, multistep process that requires sequencing the tumor’s DNA or RNA, using computational tools to identify candidate neoantigens, and manufacturing a unique product for each person. Such complexity makes it a resource-heavy undertaking, one that – for now – is feasible only at well-funded institutions[5]. The time from biopsy to complete vaccination doses is also much longer, which raises a serious concern for patients with fast-progressing disease. Moreover, many tumors are of mixed category; if some cell populations lack the targeted neoantigen, they may escape the immune response and continue to grow. All of this leads to a pressing ethical question: how can we ensure fair access? Also, such advanced therapies risk remaining out of reach in many low- and middle-income countries[6].

Several essential steps are required to realize the full potential of these vaccines: integration of artificial intelligence and machine learning in the neoantigen selection process to improve predictive accuracy and reduce development timelines. Concurrently, to overcome the tumor heterogeneity, multivalent vaccines are required to target a broader array of neoantigens, thereby offering a promising strategy. Above all, we must continue to test these vaccines with checkpoint inhibitors and other immune-modulating agents. It is also necessary to expand clinical trials by including more diverse patient groups worldwide so that benefits are broadly generalizable and access is equitable.

While still mainly in the experimental stage, personalized cancer vaccines point toward a new direction in cancer care. How we navigate the challenges of cost, production, and fairness will determine whether they can transition from compelling innovation to standard, accessible treatment.

## Data Availability

Not applicable.
